# Anammox bacterial abundance and diversity in different temperatures of purple paddy soils by ^13^C-DNA stable-isotope probing combined with high-throughput sequencing

**DOI:** 10.3389/fmicb.2023.1098681

**Published:** 2023-01-23

**Authors:** Zijie Yu, Xinhua He, Zhitong Li, Shuang Zhou, Dalu Guo, Hao Pu, Hongyan Luo

**Affiliations:** College of Resources and Environment, Southwest University, Chongqing, China,

**Keywords:** anaerobic ammonium oxidation, DNA stable-isotope probing, Illumina MiSeq, temperatures, community

## Abstract

**Introduction:**

Anaerobic ammonium oxidation (anammox) plays a vital role in the global nitrogen cycle by oxidizing ammonium to nitrogen under anaerobic environments. However, the existence, abundance, and diversity of anammox bacteria between different temperatures are less studied, particularly in purple paddy soils.

**Methods:**

^13^C-DNA stable-isotope probe combined with Illumina MiSeq high-throughput sequencing was employed to explore soil abundance and diversity of anammox bacteria. In doing so, 40–60 cm depth soils from typical purple paddy soils in Chongqing, southwest China, were cultured under ^12^CO_2_-labeled and ^13^CO_2_-labeled at 35°C, 25°C, 15°C, and 5°C for 56 days.

**Results and Discussion:**

Anammox bacteria were not labeled at all by ^13^CO_2_ at 5°C. The highest abundance of anammox bacteria was found at 25°C (3.52 × 10^6^~3.66 × 10^6^ copies·g^−1^ dry soil), followed by 35°C and 15°C (2.01 × 10^6^~2.37 × 10^6^ copies·g^−1^ dry soil) and almost no increase at 5°C. The relative abundance of *Candidatus Jettenia* sp. was higher at 25°C and 15°C, while *Candidatus Brocadia* sp. was higher at 35°C and 5°C. Our results revealed differences in anammox bacteria at different temperatures in purple paddy soils, which could provide a better understanding of soil N cycling regulated by anammox bacteria.

## 1. Introduction

According to free energy change, the process of NH_4_^+^ oxidation with NO_2_^−^ and NO_3_^−^ as electron acceptors in nature was predicted four decades ago (Broda, 1977). NH_4_^+^ combines with NO_3_^−^ to form N_2_, in which 5 moles of NH_4_^+^ requires 3 moles of NO_3_^−^ could be oxidized to 4 moles of N_2_ (Mulder et al., 1995). Twenty years ago, Strous et al. (1999) proved the existence of oxidizing ammonia to nitrogen using nitrite as an electron acceptor in a denitrification fluidized bed reactor and named this process anaerobic ammonia oxidation or anammox. Anammox bacteria were detected in a permanently flooded fallow ravine paddy field, freshwater, and sludge using ^13^C or ^15^N isotope labeling techniques (Zhou et al., 2016; Yang Y. et al., 2017; Yang X.-R. et al., 2017; Zhang et al., 2018; Li et al., 2019). The contribution of anammox to the nitrogen cycle varies significantly in different ecological environments (Zhu, 2020).

Anammox and its microorganisms play a vital role in the nitrogen cycle in farmland ecosystems (Shan et al., 2016). Anammox microorganisms belong to Gram-negative bacteria and have three components: paryphoplasm, riboplasm, and anammoxosome (Li et al., 2011). Anammoxosome, the place for anammox reaction, is a unique organelle of anammox bacteria (Li et al., 2011). Anammox bacteria can use CO_2_ or carbonate as the only carbon source, oxidize NH_4_^+^ by using NO_2_^−^ to obtain energy, and fix carbon dioxide through acetyl-CoA (Strous et al., 1999; Schouten et al., 2004). Genes encoding hydrazine synthase (*hzs*) is a critical enzyme in anammox reactions (Ali et al., 2015). *Hzs* catalyzes the synthesis of hydrazine from ammonium nitrogen and nitric oxide, which is unique to anammox metabolism (Harhangi et al., 2012; Gu et al., 2017). The genes encoding hydrazine synthase B-subunit (*hzs*B) of anammox bacteria are responsible for compiling a subunit of this enzyme (Gu et al., 2017). Using it as a biomarker of anammox bacteria can more comprehensively show the community diversity of anammox bacteria. A total of 5 anammox bacteria have been detected, including *Candidatus Brocadia, Candidatus Kuenenia*, *Candidatus Jettenia*, *Candidatus Anammoxoglobus*, and *Candidatus Scalindua* (Li Q. et al., 2018; Li N. et al., 2018). *Candidatus Scalindua* usually dominates in intertidal and deep-sea surface sediments (Fernandes et al., 2016; Wu et al., 2019), while *C. Brocadia* and *Candidatus Kuenenia* are mainly distributed in the environments such as freshwater (Yang Y. et al., 2017; Yang X.-R. et al., 2017). Paddy soils hold similar habitats to freshwater and wetland, and fertilization in paddy soils provides a suitable environment for anammox bacteria (Long et al., 2013). Purple paddy soils are characterized by shallow development, loose structure, low nitrogen content, weak nitrogen retention ability, and substantial nitrate leaching (Wu et al., 2011). Due to the unique water management measures, purple paddy soils’ anammox bacterial community composition showed many differences compared with other cultivated soils. Therefore, exploring the anammox process in purple paddy soils will enhance our further understanding of the nitrogen cycle.

Temperature is a vital factor for anammox bacterial growth and metabolism. The optimal temperature for anammox bacterial growth is 25°C–30°C (Zheng, 2001; Bai et al., 2015). Studies have shown that the specific growth rate of anammox bacteria decreased by 30%–40% with every 5°C decreases (Zheng, 2001). Wang Y. et al. (2012) and Wang S. et al. (2012) observed that the diversity of anammox bacteria was greater in warm than cold in the Pearl River Estuary sediments. Shan et al. (2018) also found that anammox bacteria were distributed in paddy fields under 5°C–35°C. The activity of anammox bacteria showed an apparent upward trend when the soil temperature increased from 15°C to 35°C. Soil oxygen concentration that decreased with the increase in soil depth significantly affected microbial community dynamics (Ratering and Schnell 2000). Both Wang Y. et al. (2012), Wang S. et al. (2012), and Wang et al. (2017) reported that the abundance of anammox bacteria in paddy soil was relatively higher at 40–60 cm than at 0–30 cm depth. Anammox bacteria seemed to prefer to live in particular niches at a particular soil depth (Humbert et al., 2010; Wang et al., 2017).

DNA-based stable-isotope probing (DNA-SIP) has been widely applied to study microbial-driven biogeochemical functioning processes at the molecular level in complex environments (Radajewski et al., 2003), link the specific metabolic function to microbial identification (Pan et al., 2018; Li et al., 2019). For instance, DNA-SIP experiments have proved anammox reactions in paddy soils under different fertilization states but not at different temperatures (Pan et al., 2018).

Hence, we investigated anammox bacterial abundance and diversity in different temperatures (35°C, 25°C, 15°C, and 5°C) of purple paddy soils. In this study, quantitative polymerase chain reaction (qPCR), high-throughput sequencing, and ^13^C-DNA stable-isotope probing were used to determine differences in the abundance and diversity of anammox bacteria at different temperatures. Community composition and diversity of anammox bacteria were analyzed by Illumina MiSeq high-throughput sequencing targeting anammox bacteria *hzs*B gene. DNA-SIP technique was used to detect the metabolic process of anammox bacteria in purple paddy soils cultured at different temperatures. Thus, the differences in anammox between these four temperatures were analyzed to potentially link the significance of anammox to nitrogen transformation deviations in the anammox bacteria community.

## 2. Materials and methods

### 2.1. Sample collection

Purple paddy soils were collected from Beibei (29°48′36″N, 106°24′33″E) in Chongqing, southwest China. The sample site has a subtropical humid monsoon climate, with a mean annual temperature and rainfall of 18.3°C and 1,086 mm, respectively. The soil’s average temperature was detected to be 25°C–32°C in summer and 10°C–13°C in winter, which were planted with rice from May to August and fallow from September to April. Purple paddy soils at 40–60 cm depth were collected vertically using the ‘YH-70 power (gasoline) soil sampler (Shaoxing, Zhejiang)’. Each soil sample was sampled using the five-point method and repeated three times to avoid pollution. After the removal of debris, air dried and then sieved (1 mm) soils. One part of the soil determined the basic physiochemical properties, and the other was used for culture experiments. The basic physiochemical properties: pH 7.38, soil temperature 26.13°C, NH_4_^+^-N 9.87 mg·kg^−1^, NO_3_^−^-N 37.12 mg·kg^−1^, NO_2_^−^-N 2.98 mg·kg^−1^, soil organic matter 33.28 g·kg^−1^, redox potential 217.24 mV, total nitrogen 1.01 g·kg^−1^, total phosphorus 0.51 g·kg^−1^, total kalium 17.41 g·kg^−1^.

### 2.2. DNA extraction, real-time quantitative PCR and DNA-SIP culture

According to the manual procedures, DNA was extracted from the cultivated soils using a FastDNA^®^ Spin Kit for Soil (MP Biomedicals, Cleveland, OH, United States). A NanoDrops 2000 UV–Vis Spectrophotometer instrument (NanoDrop Technologies, Wilmington, DE, United States) was used for detecting the *hzs*B gene copy numbers. Quantitative PCR was used to detect the anammox bacterial abundances. Using fluorescent dye SYBR-Green, an ABI QuantStudio™ 6 Flex system real-time PCR instrument (Applied Biosystems, CA, United States) detected anammox bacterial abundances. The *hzs*B gene of anammox bacteria was targeted by HSBeta396F/HSBeta742R primers (5′-ARGGHTGGGGHAGYTGGAAG-3′ and 5′- GTYCCHACRTCATGVGTCTG-3′; Wang Y. et al., 2012; Wang S. et al., 2012). Amplification was performed in 20 μL reaction mixtures, where DNA template 2 μL, TB Green^®^Premix Ex Taq (Tli RNaseH Plus, Takara, Dalian, China) 10 μL, forward primer 1 μL (10 μM), reverse primer 1 μL (10 μM), filling the rest with ddwater. The reaction temperature and time of *hzs*B amplification: 95°C with 3 min; the 40 cycles of 95°C with 30 s, 59°C with 30 s, and 72°C with 30 s (Li et al., 2019). Standard curves were obtained with 10-fold serial dilutions of the plasmid DNAs. Our results’ correlation coefficient and efficiency are above 0.99% and 98%, respectively (Table 1).

**Table 1:** The DNA concentration of purple paddy soils in different treatments.

Different temperatures	Treatment	Concentration (ng•μL^−1^)	OD_260_/OD_280_
35°C	0d	54.9	1.89
	56d ^12^CO_2_	57.1	1.88
	56d ^13^CO_2_	56.8	1.98
25°C	0d	54.9	1.89
	56d ^12^CO_2_	55.6	1.86
	56d ^13^CO_2_	58.2	1.91
15°C	0d	54.9	1.89
	56d ^12^CO_2_	49.7	1.90
	56d ^13^CO_2_	50.6	1.88
5°C	0d	54.9	1.89
	56d ^12^CO_2_	42.7	1.90
	56d ^13^CO_2_	44.3	1.91

Purple paddy soil slurries were made with 10 g fresh soils and helium (He)-purged water in 100-mL glass bottles, adjusting to 60% of the maximum field water holding capacity of the purple paddy soils (Shan et al., 2018; Liu et al., 2019). A pre-culture experiment was performed before conducting soil DNA-SIP micro-universe culture to study anammox. Before pre-culture, 5 min N_2_ was injected into each bottle, kept the bottle in anaerobic. Pre-culture for 2 weeks at different temperatures to reduce soil CO_2_ emission, NH_4_^+^, NO_3_^−^, NO_2_^−^, and dissolved oxygen (Xiong et al., 2022). After pre-culture, the accumulated CO_2_ in the bottle will be discharged, and the weekly emission of CO_2_ in the glass bottles (cumulative amount of 7 days) will be less than 0.5% (Xiong et al., 2022). The DNA-SIP micro-universe culture consisted of different treatments:^12^CO_2_-labeled and ^13^CO_2_-labeled at 35°C, at 25°C, at 15°C, and at 5°C (CO_2_ purity ≥ 99%, CO_2_ abundance ≥ 99%, 5 L gas, Wuhan Isotope Technology Co., Ltd), respectively. 5 mL CO_2_ was added into the glass bottles using a syringe regularly every week, and the volume fraction of CO_2_ was adjusted to 5%. Destructive sampling was performed at 0-day and 56-day, with 3 replicates per treatment. For DNA-SIP analysis, about 3 μg of soil total DNA was mixed with 1.725 g·mL^−1^ CsCl in a 5.1 mL ultra-high speed centrifuge tube (Jia and Conrad 2009). The Beckman vertical centrifugal rotor Vti65.2 was centrifuged at 20°C for 44 h at 45,000 r·min^−1^ (190,000*g*, Beckman Coulter, Palo Alto, CA, United States). After the ultra-high speed separation, the stratified DNA recovery began. Replacement liquid (sterilized water) was injected at a constant flow rate in the upper part of the centrifuge tube, and 15 layered samples of equal volume and different densities were collected in the lower part of the sterilized centrifuge tube. The buoyancy density and the refractive index of each layer solution were calculated (*ρ* = −75.9318 + 99.2031*x*-31.2551*x*^2^, *ρ* denotes the buoyant density, *x* denotes the refractive index).

### 2.3. PCR and high-throughput sequencing analysis

PCR analysis of *hzs*B gene was performed using modified primers HSBeta396F/HSBeta742R with some modifications (Wang Y. et al., 2012; Wang S. et al., 2012). 20 μL reaction system was used in the PCR analysis (TransStart Fastpfu DNA Polymerase), including 5 × FastPfu Buffer 4 μL, 2.5 mM dNTPs 2 μL, forward primer (5 μM) 0.8 μL, reverse primer (5 μM) 0.8 μL, FastPfu Polymerase 0.4 μL, BSA 0.2 μL, template DNA 10 ng, and ddwater. PCR reaction parameters: a. 1× (3 min at 95°C); b. 40 of cycles × (30 s at 95°C; 30 s at 59°C; 45 s at 72°C); c. 10 min at 72°C, 10°C. All PCR reactions were amplified in triplicate. PCR products were extracted from 2% agarose gel. The AxyPrep DNA gel extraction kit (Axygen Biosciences, Union City, CA, United States) was purified according to the manufacturer’s instructions and quantified using a Quantus™ Fluorometer (Promega, United States). According to the standard protocol of Majorbio Bio-Pharm Technology Co. Ltd. (Shanghai, China), the purified amplicons were mixed in equimolar amounts and paired for sequencing on the Illumina MiSeq PE300 platform (Illumina, San Diego, United States). The sequence data were submitted to the NCBI Sequence Read Archive (SRA) database with the accession numbers SRP407824.

### 2.4. Statistical analysis

The sequencing data were analyzed using the Majorbio Platform, Shanghai. Using Chromas, NCBI Blast,^1^ and Mega v11.0 to analyze gene sequences. All the high-quality recovered sequences were blasted in the NCBI database. Mothur software was used to group sequences with 97% similarity into the same operational classification unit (OTU). The phylogenetic tree of *hzs*B gene sequence was constructed with the NJ (neighbor-joining) method through Mega v11.0. An Origin 2022 (OriginLab Corp., Northampton. MA, United States) were used to plot the contents of ammonium, nitrate nitrogen and the abundance of anammox bacteria. One-way analysis of variance (ANOVA) was used to analyze soil samples in SPSS25.0.

## 3. Results

### 3.1. Soil ammonium nitrogen and nitrate nitrogen in different treatments

**Figure 1:**
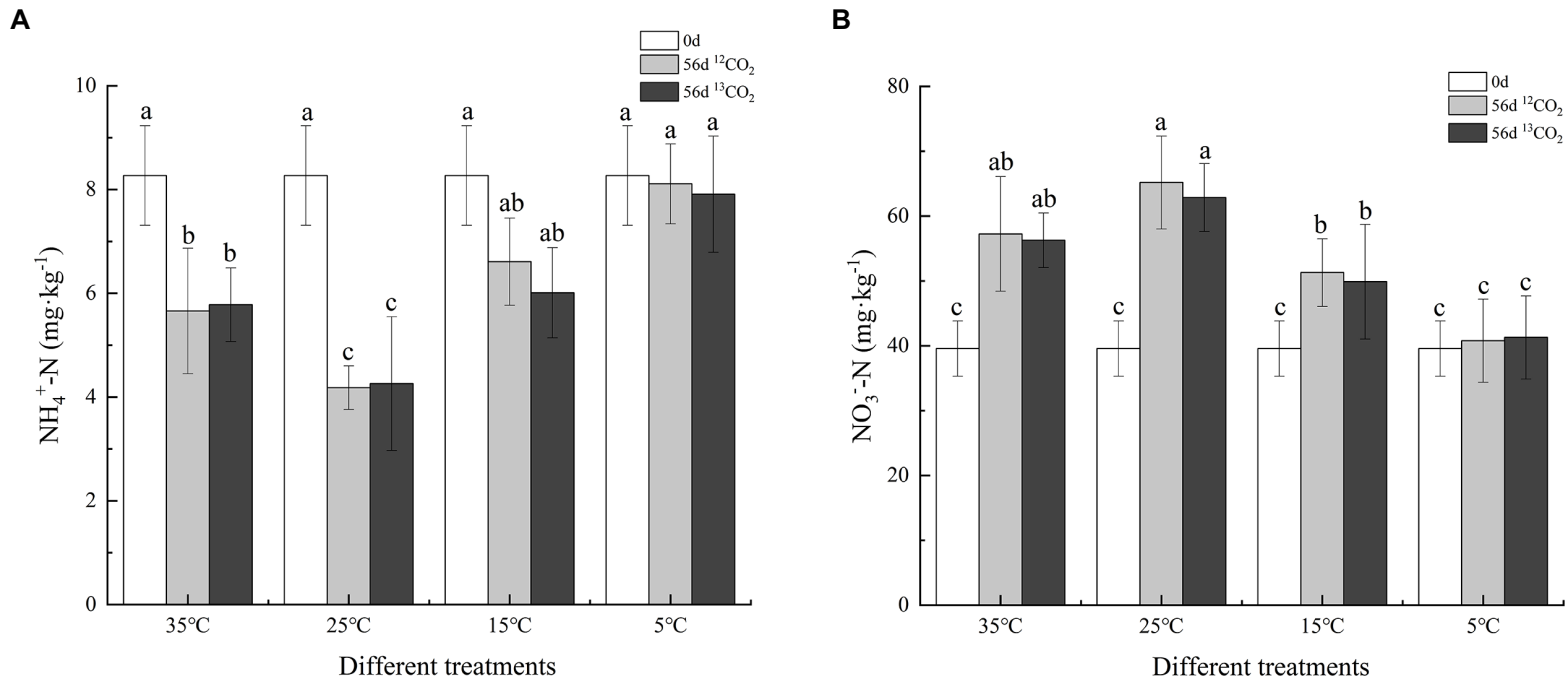
Variations in soil ammonium nitrogen **(A)** and nitrate nitrogen **(B)** after 56 days cultured in different treatments. Different letters mean significantly differences among the treatments (*p ≤* 0.05, *n* = 3).

The content of soil ammonium nitrogen and nitrate nitrogen changed under different temperatures after 56 days cultured in Figure 1. The content of soil ammonium nitrogen decreased significantly at 35°C, 25°C, and 15°C, but not at 5°C (*p* < 0.05). The decrease was most significant at 25°C, with the consumption rates of soil ammonium nitrogen in ^12^CO_2_-labeled and ^13^CO_2_-labeled, reaching 49.46% and 48.49%, respectively. At 35°C, soil ammonium consumption rates were 31.56% and 30.11%, respectively, slightly higher than 15°C (20.07% and 27.33%). The soil nitrate nitrogen trend was opposite to soil ammonium nitrogen. After 56 days cultured, the soil nitrate nitrogen content increased, and the most significant growth rates were 64.74% and 58.90% at 25°C, followed by 44.72% and 42.24% at 35°C, and finally, 29.63% and 26.09% at 15°C, while there was no significant change at 5°C.

### 3.2. Labeled determination of anammox bacteria in different treatments

**Figure 2:**
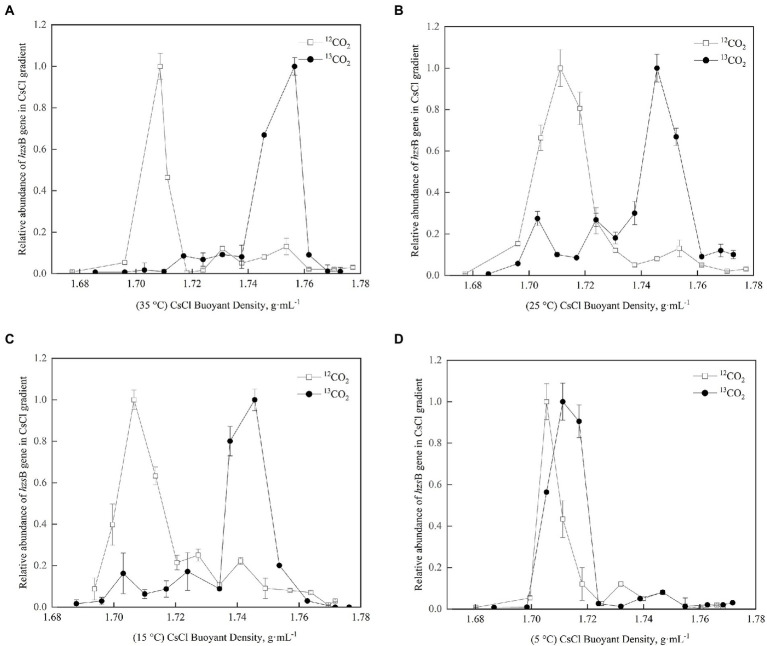
Quantitative distribution of the anammox bacteria hzsB genes across the entire buoyant density gradient of the DNA fractions in different treatments. **(A)** is 35°C; **(B)** is 25°C; **(C)** is 15°C; **(D)** is 5°C. Different letters mean significant differences among the treatments (*p* ≤ 0.05, n = 3).

According to the buoyancy density of DNA, ^13^C-DNA was separated from ^12^C-DNA in purple paddy soils in different treatments, and the *hzs*B gene of anammox bacteria was quantified by fluorescence quantitative PCR. In Figures 2A–C, the anammox bacteria treated with ^12^CO_2_-labeled were all in a single peak, all in lighter densities (1.70–1.72 g·mL^−1^). In comparison, the anammox bacteria treated with ^13^CO_2_-labeled had a peak in the heavier densities (1.74–1.76 g·mL^−1^), indicating that the functional gene of *hzs*B was successfully labeled in the culture process. The results indicated that anammox reaction processes existed in 35°C, 25°C, and 15°C of purple paddy soils. While in Figure 2D, ^12^CO_2_-labeled and ^13^CO_2_-labeled were all in the lighter densities, indicating that anammox bacteria may not be involved in metabolism.

### 3.3. Abundance of anammox bacteria in different treatments

**Figure 3:**
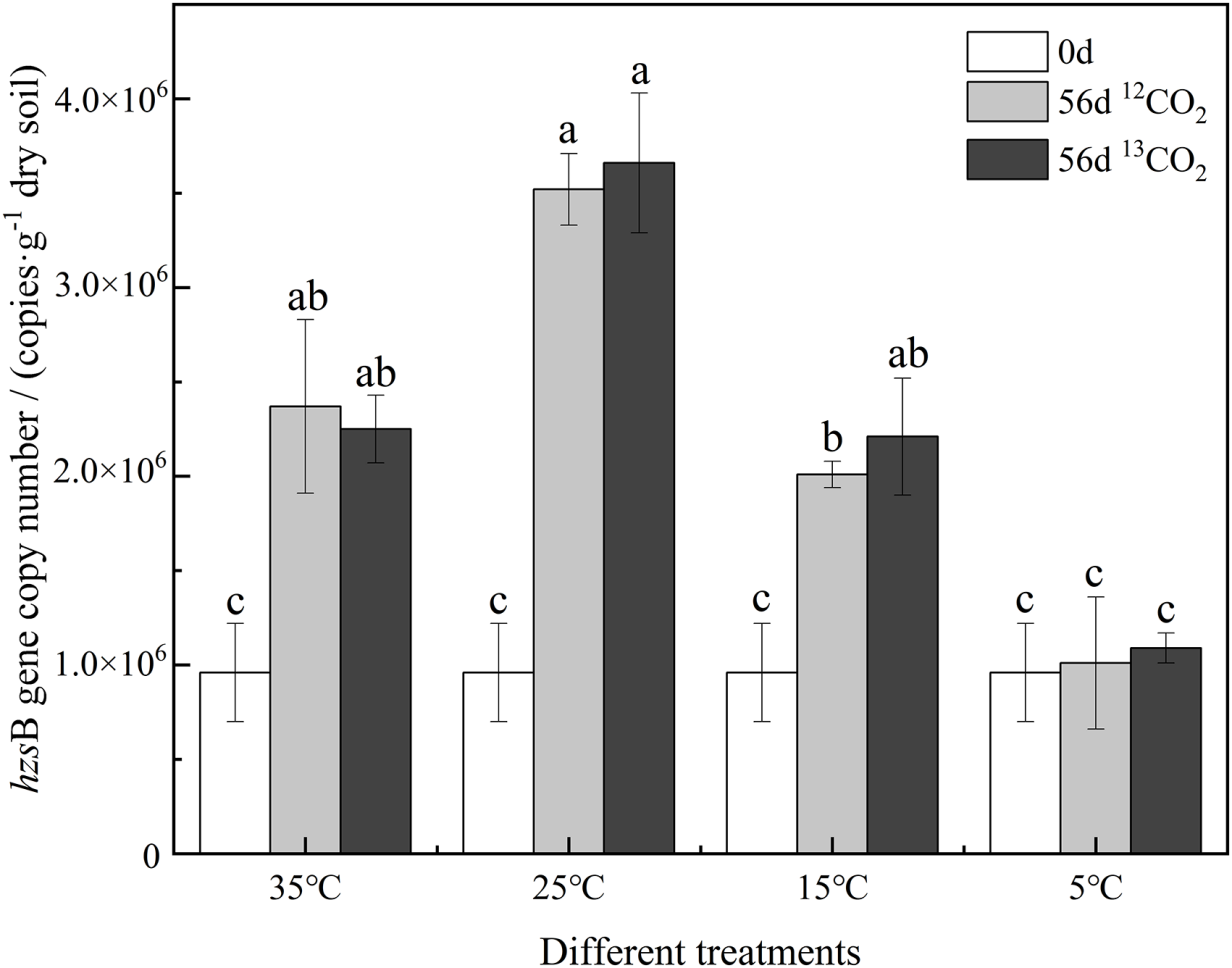
The copy number of *hzs*B genes of anammox bacteria in different treatments. Different letters mean significantly differences among the treatments (*p ≤* 0.05, *n* = 3).

The *hzs*B gene of samples cultured for 0-day and 56-day was analyzed by qPCR to determine the abundance of anammox bacteria in Figure 3. In the culture at different temperatures, the abundance of anammox bacteria showed significant differences. On 0-day, the abundance of anammox bacteria was 9.6 × 10^5^ copies·g^−1^ dry soil. After 56 days of cultured, the abundance of anammox bacteria increased significantly at 25°C, a maximum of 3.52–3.66 × 10^6^ copies·g^−1^ dry soil (*p ≤* 0.05). Anammox bacteria increased significantly at 35°C and 15°C, and their abundance reached 2.01–2.37 × 10^6^ copies·g^−1^ dry soil. In soil cultured at 5°C, there was no significant change in the abundance of bacteria labeled with ^12^CO_2_ and ^13^CO_2_.

### 3.4. Community structure of anammox microorganisms after culture

**Figure 4:**
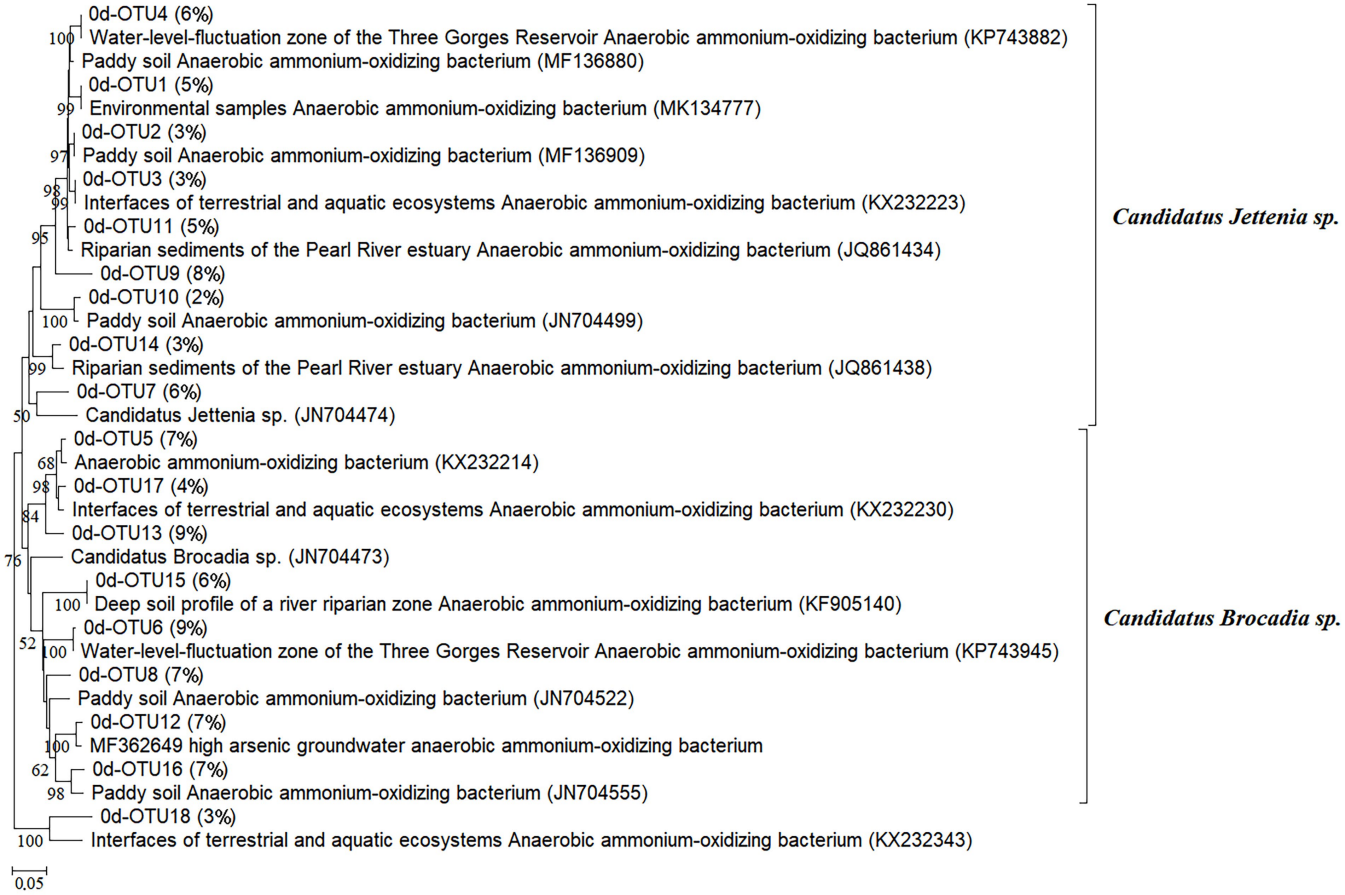
Phylogenetic analysis of the anammox bacterial *hzs*B genes at 0-day of culture in purple paddy soils.

**Figure 5:**
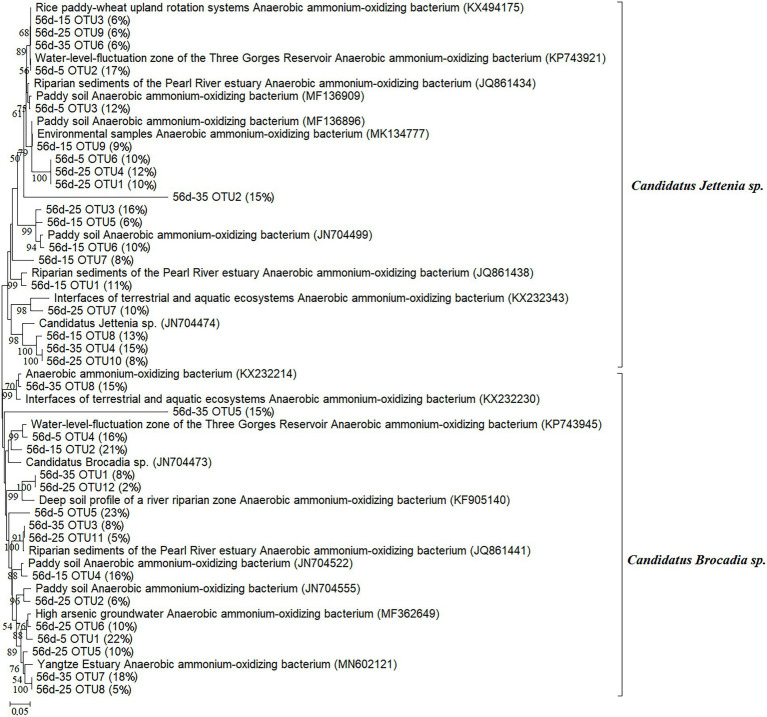
Phylogenetic analysis of the anammox bacterial *hzs*B genes from the ^13^CO_2_-labeled microcosms at 56 days of culture in purple paddy soils. “56d-15 OTU3 (6%)” means OTU3 accounted for 6% at 15°C for 56 days.

After 56 days of culture, the purple paddy soils were stratified with ^12^CO_2_-labeled and ^13^CO_2_-labeled in different treatments. Illumina MiSeq high-throughput sequencing based on *hzs*B functional genes of anammox bacteria was performed on the obtained labeled heavy-layer DNA (^13^CO_2_-labeled). Phylogenetically analyzed the anammox bacterial *hzs*B genes at 0-day and 56-day in different treatments of purple paddy soils. In 0-day, a total of 18 OTUs were phylogenetically affiliated to *C. Brocadia* sp. and *C. Jettenia* sp., with the relative abundance of *C. Brocadia* sp. accounting for 56% and *C. Jettenia* sp. accounting for 41% (Figure 4). After 56 days of culture at different treatments, 8, 11, 9, and 6 OTUs were detected at 35°C, 25°C, 15°C, and 5°C, respectively. At 25°C and 15°C, the relative abundance of *C. Brocadia* sp. decreased to 38% and 37%, and *C. Jettenia* sp. increased to 62% and 63%. While at 35°C and 5°C, the relative abundance of *C. Brocadia* sp. increased to 64% and 61% (Figure 5).

## 4. Discussion

In this study, the variation of anammox bacterial community abundance and diversity with different temperatures in purple paddy soils were presented by *hzs*B gene. ^13^C-labeled presenting in the anammox bacteria indicated that anammox bacteria were involved in the anammox reaction at 35°C, 25°C, and 15°C (Figure 2). The results provide evidence for anammox reaction in purple paddy soils at different temperatures. After 56 days cultured, soil ammonium nitrogen decreased, while nitrate nitrogen and the abundance of anammox bacteria increased at different temperatures. The essential factors affecting anammox bacterial activity in waterlogged ecosystems may have NO_2_^−^, NO_3_^−^, and soil oxygen content (Shen et al., 2013). Anoxic and waterlogged conditions in cultured soil at different treatments could provide a more suitable environment for anammox bacteria than upland soils. Under *in situ* conditions, oxygen differs between the topsoil (411 mV–484 mV) and lower depth (234 mV–383 mV) in paddy soil, which adversely affects anammox activity (Bai et al., 2015), so the deeper soils were used in this study. Shan et al. (2016) found that the relatively higher soil total nitrogen content (1.87–2.47 g·kg^−1^) could promote anammox bacterial growth at a detectable level. Similarly, Zhu et al. (2011) detected anammox bacteria in fertilized rice fields. These results may indicate that the widespread distribution of anammox bacteria in terrestrial ecosystems is mainly related to soil nitrogen content.

The abundance and proportion of anammox bacteria in purple paddy soils under different temperatures were different (Figures 3–5). The results found that after culturing the soil internal detected *C. Brocadia* sp. and *C. Jettenia* sp., speculated that, one reason, some heterotrophic and simple organic compounds produced by microbial metabolism accumulated in the late time, such as formic acid, acetic acid, propionic acid (Ali et al., 2015; Oshiki et al., 2016). Different anammox bacteria absorb these simple organic acids for their growth (Wang et al., 2018). For example, the growth competitiveness was enhanced by formic acid or acetic acid for *C. Brocadia* sp. (Oshiki et al., 2016), while by propionic acid for *C. Jettenia* sp. (Ali et al., 2015). Another reason is that anammox diversity is affected by inorganic nitrogen fertilization in agricultural fields (Yang et al., 2015). For instance, the application of ammonium and nitrate nitrogen based fertilizers, not changing temperatures, can alter the composition of anammox bacterial community structures (Yang Y. et al., 2017; Yang X.-R. et al., 2017). A continuous N-fertilization may have allowed *C. Brocadia* sp. and *C. Jettenia* sp. to be adapted and predominate in such nitrogen-rich agricultural soils (Long et al., 2013).

Changes in external temperature could directly or indirectly affect the community composition of anammox bacteria (Shan et al., 2018). In a study of anammox bacteria in the sediments of the Pearl River Estuary, Wang Y. et al. (2012) and Wang S. et al. (2012) found that the abundance of anammox bacteria in the sediments of river ports was greater in cold temperatures, while the opposite was true for the diversity of anammox bacteria. Anammox showed the highest activities at 15°C and was undetectable at 37°C (Shan et al., 2018). These results implying different anammox bacterial species have different sensitivity to temperature changes in the environment, showing that temperature is a principal environmental factor to shape the diversity, abundance, and distribution of anammox bacteria (Yang Y. et al., 2017; Yang X.-R. et al., 2017; Wu et al., 2020; Chen et al., 2022). The temperature may also exhibit the significance of the biogeographic distribution of anammox bacterial community structure and diversity (Shan et al., 2018). Our study’s results also show that different temperatures affected the abundance and diversity of anammox bacteria (Figure 2). The abundance was higher in warm temperatures (i.e., 35°C and 25°C) and lower in cold temperatures (i.e., 5°C; Figure 3). Shan et al. (2018) found that a noticeable trend of anammox bacterial activity, first rising and then falling, when the ambient temperature increased from 15°C to 35°C, reaching the highest at 25°C in paddy soil, consistent with our results (Figures 2, 3). As a novel biomarker for the molecular detection of anammox bacteria, the *hzs*B genes of anammox bacteria were abundant in 30–60 cm deep layers than at 0–30 cm layer, with the higher abundance of 2.79 × 10^6^ copies·g^−1^ dry soil at 40–50 cm (Wang Y. et al., 2012; Wang S. et al., 2012), about 5.42 × 10^4^ copies·g^−1^ dry soil at the surface layer (Xu et al., 2017). In our purple paddy soils, the abundance of *hzs*B genes was 9.6 × 10^5^ copies·g^−1^ dry soil at 40–60 cm depth. After 56 days cultured, the abundance of anammox bacteria increased at 35°C, 25°C, and 15°C, reaching 2.01 × 10^6^–3.66 × 10^6^ copies·g^−1^ dry soil (Figure 3), while not in 5°C, which were similar to the results of Wang Y. et al. (2012) and Wang S. et al. (2012), lower than that in the Pearl River Estuary sediment (Wu et al., 2020) and coastal wetland (Hou et al., 2015). The reason may be that the nutrient (e.g., NH_4_^+^, NO_2_^−^/NO_3_^−^ and SOM) content of purple paddy soils was lower than that of sediment and wetland, and low temperature was also not conducive to microbial growth and metabolism (Wang Y. et al., 2012; Wang S. et al., 2012; Bai et al., 2015). The optimal temperature of anammox depends not only on the ambient temperature, but also on the adaptive capacity of anammox bacteria (Shen et al., 2017; Tomaszewski et al., 2017), which are less adaptable at lower temperatures (5°C).

High-throughput sequencing has a higher coverage of microbial diversity and could detect abundant and rare microorganisms in the environments. Moreover, it can create a more extensive data set and provide a more comprehensive insight into the analysis of anammox bacteria community structure while more widely used than the clone libraries (Shen et al., 2017; Hong et al., 2019; Yang et al., 2020). The *hzs*B gene was phylogenetically affiliated to *C. Brocadia* sp. and *C. Jettenia* sp. (Figures 4, 5). The relative abundance of anammox bacteria varied in different temperatures (Figure 5). This anammox genus (i.e., *C. Brocadia*) can obtain energy from short-chain organic acids and use alternative electron donors to adapt better to different environments (Long et al., 2013; Yang et al., 2015). Studies have also shown that *C. Brocadia* may have good adaptability in paddy soils, and the detection frequency of *C. Brocadia* and *C. Kuenenia* is significantly higher than *C. Jettenia* (Zhu et al., 2011; Shan et al., 2016). In contrast, Long et al. (2013) found *C. Jettenia* was a higher abundance in agricultural soils, which is consistent with our results. The soils collected by Long et al. (2013) were deeper, compared with Shan et al. (2016) and Zhu et al. (2011), and fertilized in the last 5 years, so there may have been more *C. Jettenia*. Compared with freshwater ecosystems (Yang Y. et al., 2017; Yang X.-R. et al., 2017; Zhu, 2020), the diversity and abundance of anammox bacteria in paddy soil were higher. Anammox bacteria have the specific characteristics of metabolic diversification when growing in heterogeneous soil that can provide a favorable habitat for their growth and function (Sonthiphand et al., 2014). Various anammox bacteria can be detected in agricultural soils, with the most common categories being *C. Brocadia*, *C. Jettenia*, and *C. Kuenenia*. In our study, *C. Brocadia* sp. and *C. Jettenia* sp. were detected, but *C. Kuenenia* was not, which may be related to soil parent material (Long et al., 2013). However, our study was inconsistent with the results of anammox bacteria identified by Kwok-Ho Lee et al. (2014) as *Candidatus Scalindula* in 6 m depth under soil fertilization reduces the community diversity of anammox bacteria in purple paddy soil. *Candidatus Scalindula* is often detected in places with high salinity (Fernandes et al., 2016; Wu et al., 2019).

## 5. Conclusion

We revealed the feasibility of using ^13^C-DNA stable-isotope probe combined with the Illumina MiSeq high-throughput sequencing technique for analyzing anammox in different temperatures of purple paddy soils. Anammox bacteria grew more suitable at 25°C. Phylogenetic tree analysis showed that the dominant anammox bacteria were *C. Brocadia* sp. and *C. Jettenia* sp., which abundance and community structures were different in different temperatures.

## Data availability statement

The data presented in the study are deposited in the NCBI repository, accession numbers SRR22279417-SRR22279430.

## Author contributions

ZY, ZL, DG, and HL conceived and designed the experiments. ZY, SZ, DG, and HP performed the experiments. ZY, XH, ZL, SZ, DG, HP, and HL wrote and revised the manuscript. All authors contributed to the article and approved the submitted version.

## Funding

This work was supported by the National Natural Science Foundation of China (41301315), Fundamental Research Funds for the Central Universities (XDJK 2019B072), the Natural Science Foundation of Chongqing Municipality (Cstc2012JJA80024 and cstc2019jcyj-msxmX0304).

## Conflict of interest

The authors declare that the research was conducted in the absence of any commercial or financial relationships that could be construed as a potential conflict of interest.

## Publisher’s note

All claims expressed in this article are solely those of the authors and do not necessarily represent those of their affiliated organizations, or those of the publisher, the editors and the reviewers. Any product that may be evaluated in this article, or claim that may be made by its manufacturer, is not guaranteed or endorsed by the publisher.

## Footnotes

^1^ https://www.ncbi.nlm.nih.gov/
